# Vitexin induces apoptosis and enhances daunorubicin efficacy in acute leukemia via modulation of the HIF-1α/Bcl-2/caspase-3 pathway

**DOI:** 10.1038/s41598-025-32789-y

**Published:** 2025-12-14

**Authors:** Chutiphong Jirawatpraphakorn, Dalina Tanyong, Attasak Jaree, Weerapat Owattanapanich

**Affiliations:** 1 Division of Hematology, Department of Medicine, Faculty of Medicine Siriraj Hospital, Mahidol University, Bangkok, Thailand; 2 Department of Clinical Microscopy, Faculty of Medical Technology, Mahidol University, Nakhon Pathom, Thailand; 3 Center for High-Value Products from Bioresources: HVPB, Department of Chemical Engineering, Faculty of Engineering, Kasetsart University, Bangkok, Thailand

**Keywords:** Vitexin, Apoptosis, Acute myeloid leukemia, Acute lymphoblastic leukemia, HIF-1α, Cancer, Cell biology, Drug discovery, Oncology

## Abstract

Acute leukemia is an aggressive hematologic malignancy with limited treatment success owing to drug resistance, severe adverse effects, and high costs. Vitexin, a natural compound, demonstrates promising anticancer properties by modulating multiple pathways and inducing apoptosis, while maintaining favorable toxicity profiles. This study examined the pro-apoptotic effects of vitexin on leukemic cell lines (NB-4 and MOLT-4) and patient-derived bone marrow cells, as well as its combined effect with daunorubicin. Cytotoxicity was evaluated using MTT, apoptosis was assessed via Annexin V/PI flow cytometry, and molecular mechanisms were elucidated through in silico bioinformatic, RT-qPCR, and Western blot analyses. Vitexin decreased cell viability in a dose- and time-dependent manner (48 hours of IC_50_: 901 µM in NB-4, 929 µM in MOLT-4), with minimal toxicity in normal PBMCs. Synergistic interaction with daunorubicin was confirmed through the combination index. Vitexin elevated apoptosis up to 42.82% by downregulating HIF-1α and upregulating caspase-3 at both transcriptional and translational levels. Patient-derived bone marrow cells, the combination treatment induced the highest apoptosis (22.15% AML, 18.82% ALL). Vitexin induces apoptosis via modulation of HIF-1α/Bcl-2/caspase-3 pathway and potentiates efficacy of daunorubicin, thereby supporting potential as an adjunctive therapeutic in acute leukemia. Further in vivo studies are necessary to elucidate pharmacokinetics and clinical applicability.

**Supplementary Information:**

The online version contains supplementary material available at 10.1038/s41598-025-32789-y.

## Introduction

 Leukemia is a severe hematologic malignancy characterized by the uncontrolled proliferation of abnormal white blood cells, which can originate from either the bone marrow or the lymphatic system. It remains one of the most challenging cancers to treat, due to the development of drug resistance and the severe adverse effects associated with conventional therapies. Acute leukemia encompasses acute myeloid leukemia (AML) and acute lymphoblastic leukemia (ALL), poses a significant global health burden, contributing to substantial morbidity and mortality worldwide^1–3^. Acute leukemia is marked by the accumulation of immature cells in the bone marrow, which impedes the normal hematopoiesis and leads to symptoms such as fatigue, fever, and heightened susceptibility to infections. Despite considerable advancements in treatment modalities, including chemotherapy, targeted therapy, and bone marrow transplantation, the efficacy of current advanced therapies is often limited by the emergence of chemotherapeutic drug resistance, the severity of adverse effects, and their high cost. This necessitates the urgent exploration and discovery of alternative therapeutic agents.

In recent years, there has been a growing interest in complementary and alternative medicine (CAM) as an adjunct to standard therapies. These approaches are increasingly popular in various countries where natural compounds are well-recognized and integrated into healthcare practices^4–8^. Natural compounds, such as vitexin, are gaining significant attention as potential alternative therapeutic agents in cancer treatment due to their ability to modulate multiple pathways and mechanisms involved in cancer cell proliferation and programmed cell death^6^.

Programmed cell death (PCD) is a fundamental biological process crucial for maintaining cellular homeostasis and eliminating damaged or unnecessary cells^9–11^. Among the various types of PCD, apoptosis is the most widely recognized and extensively researched. Apoptosis plays a vital role in facilitating the elimination of infected or malignant cells, thereby preventing the potential development and progression of diseases such as cancer^12,13^. However, cancerous cells frequently acquire mutations that enable them to evade normal apoptotic signals, leading to uncontrolled proliferation and disease persistence. Consequently, targeting apoptotic pathways has emerged as a key therapeutic strategy aimed at restoring the apoptotic capability of cancerous cells, thereby disrupting disease progression and enhancing the effectiveness of existing treatments^14,15^.

The development of alternative cancer treatments utilizing natural compounds has advanced considerably^5^. Among these, vitexin, a naturally occurring flavonoid glycoside found in various plants such as the mung bean (*Vigna radiata*)^16,17^, has demonstrated promising anti-cancer properties across multiple studies. Vitexin exerts its effects by modulating various signaling pathways, including the induction of apoptosis and inhibition of cell proliferation. Recent studies have shown that vitexin can induce apoptosis in diverse cancer cell lines^18–22^. Furthermore, vitexin has exhibited the ability to induce apoptosis in leukemic cells, suggesting its significant potential as an alternative therapeutic option for patients with acute leukemia^18–21,23–28^.

Daunorubicin was selected as the chemotherapeutic drug for this study because it is an anthracycline used in standard induction regimens for acute myeloid leukemia, such as the 7+3 protocol, and is also incorporated into several treatment protocols for acute lymphoblastic leukemia. Its mechanisms of action, including DNA intercalation, topoisomerase II inhibition, and induction of oxidative stress, overlap with apoptotic pathways modulated by vitexin, making this combination clinically relevant and mechanistically appropriate for the investigation^29–31^.

This study aims to comprehensively evaluate the effect of vitexin on apoptosis induction in various leukemic cell lines and primary leukemic cells obtained from patients. Additionally, it will assess the effectiveness of combining vitexin with the conventional chemotherapy drug, daunorubicin, in promoting apoptosis in leukemic cells. Finally, this research will investigate the underlying molecular mechanisms and signaling pathways involved in the apoptotic process triggered by vitexin.

## Results

### Cytotoxic effect of vitexin on leukemic cell lines

**Fig. 1:**
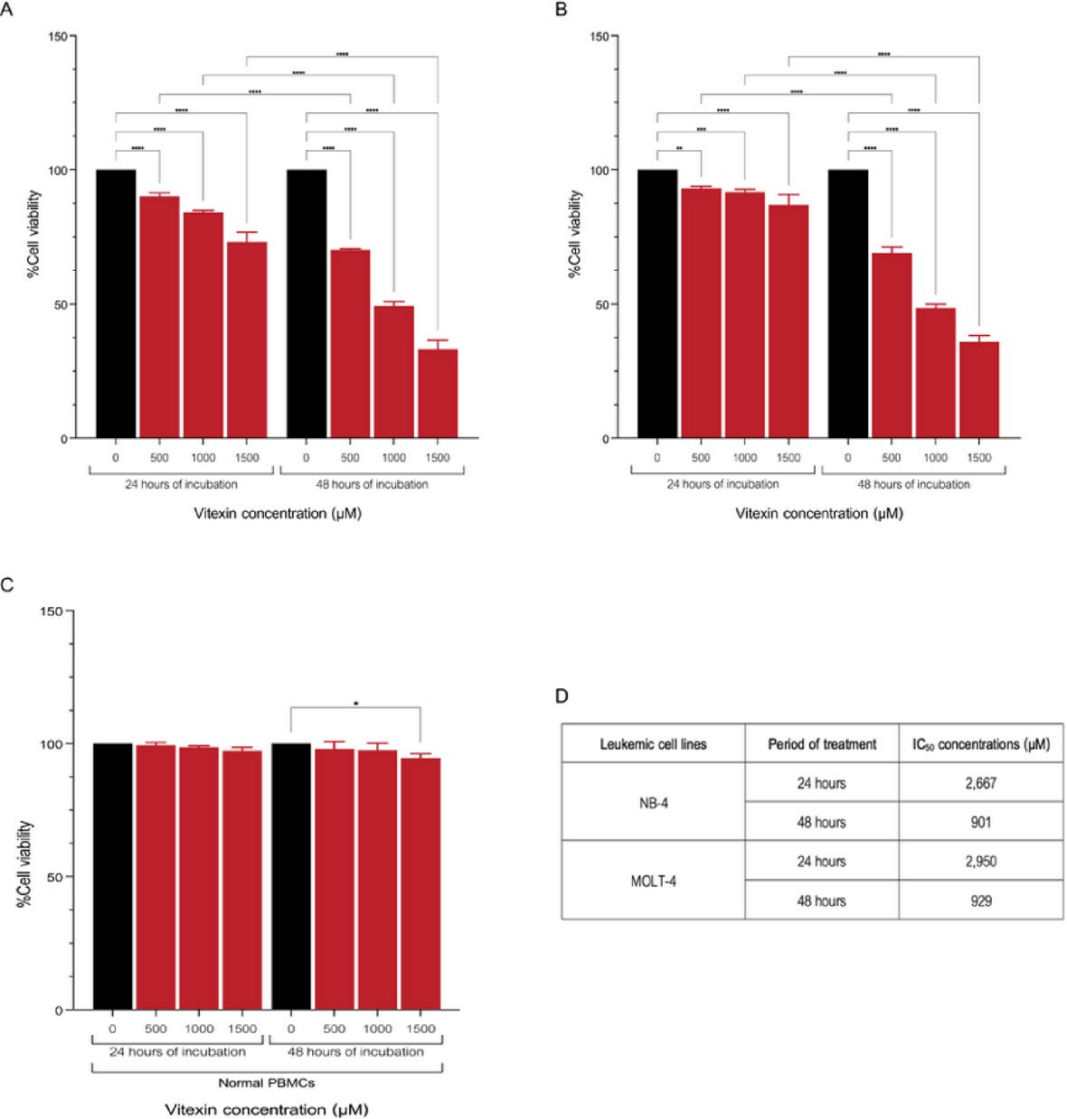
The cytotoxicity of various concentrations of vitexin was investigated in NB-4 (**A**) and MOLT-4 (**B**) cell lines, as well as in normal PBMCs (**C**), and IC_50_ concentrations of vitexin following 24 and 48 hours of treatment in both NB-4 and MOLT-4(D). Cell viability is presented as a percentage relative to the untreated control. (*p < 0.05, **p < 0.01, ***p < 0.001, ****p < 0.0001).

The cytotoxic effect of vitexin on leukemic cell lines was evaluated using the MTT assay. The findings demonstrate that vitexin significantly reduces cell viability in both NB-4 and MOLT-4 cell lines in a dose- and time-dependent manner (Fig. 1A, B). Specifically, after 24 h of treatment, the half-maximal inhibitory concentration (IC_50_) for vitexin was determined to be 2,667.00 µM in NB-4 cells and 2,950.00 µM in MOLT-4 cells. Following 48 h of treatment, the IC_50_ concentrations decreased to 901.00 µM for NB-4 cells and 929.00 µM for MOLT-4 cells (Fig. 1D). Based on these results, the 48 hours of IC_50_ concentrations for both cell lines were selected as the optimal concentrations for all subsequent experiments.

To assess the potential of vitexin for selective anti-leukemic effects and minimal toxicity to healthy cells, the impact of vitexin on normal peripheral blood mononuclear cells (PBMCs) was investigated. As shown in Fig. 1C, a statistically significant reduction in viability was observed only at a high vitexin concentration of 1,500 µM. Importantly, IC_50_ concentrations and other tested lower concentrations did not induce a significant decrease in normal PBMC viability. This indicates that the selected therapeutic concentrations of vitexin exert minimal adverse effects on healthy immune cells, supporting its potential as a safe and effective treatment strategy for leukemia.

### Combination drugs investigation

**Fig. 2:**
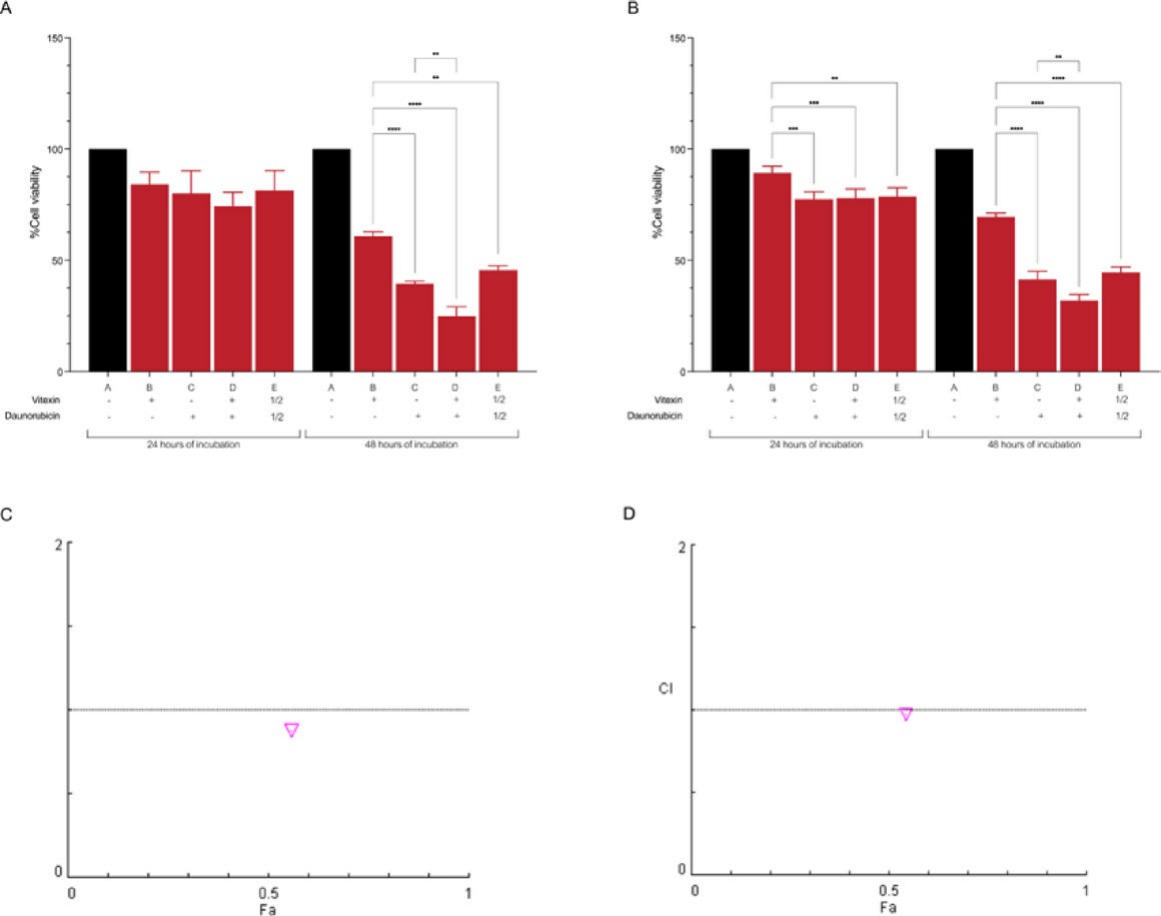
The cytotoxicity of vitexin combined with daunorubicin in NB-4 (**A**) and MOLT-4 (**B**) cells. (**A**–**E**) on the x-axis correspond to the treatment conditions used in this experiment (A = control, B = IC_50_ of vitexin, C = IC_50_ of daunorubicin, D = IC_50_ of vitexin combination with IC_50_ of daunorubicin, and E = ½ IC_50_ of vitexin combination with ½ IC_50_ of daunorubicin). Cell viability is presented as a percentage relative to the untreated control. Combination index (CI) calculation and plot for NB-4 (**C**) and MOLT-4 (**D**) with Fa, a parameter generated by CompuSyn that reflects the proportion of cell growth inhibited by each treatment. (*p < 0.05, **p < 0.01, ***p < 0.001, ****p < 0.0001).

The cytotoxicity of this combination was evaluated after 24 and 48 hours of treatment (Fig. 2A, B). The combination index (CI) was calculated to investigate the drug interaction. For the NB-4 cell line, the calculated CI was 0.8 (Fig. 2C), indicating a synergistic effect between vitexin and daunorubicin. Conversely, in the MOLT-4 cell line, the calculated CI was 1.0 (Fig. 2D), suggesting a potential additive effect when vitexin is combined with daunorubicin. These findings highlight the differential interaction of vitexin with daunorubicin across different leukemic cell lines. Based on these CI definitions, vitexin and daunorubicin demonstrated a synergistic interaction in NB-4 cells (CI = 0.8) and an additive interaction in MOLT-4 cells (CI = 1.0).

### The effect of vitexin and combination treatment on inducing apoptosis in leukemic cell lines

**Fig. 3:**
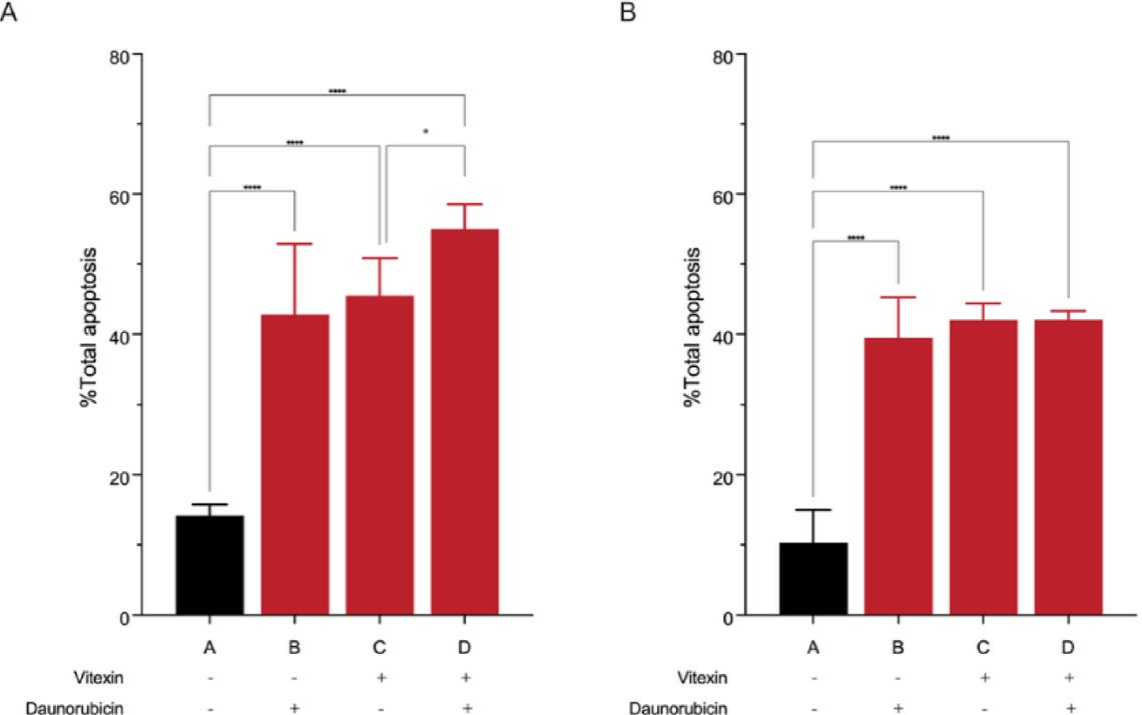
Effect of vitexin, daunorubicin, and their combination on apoptosis induction in NB-4 (**A**) and MOLT-4 (**B**) leukemic cell lines. Cells were treated with vitexin, daunorubicin, or the combination for 48 hours, and apoptosis was assessed using Annexin V-FITC/PI staining. The bar graphs show the percentage of total apoptotic cells for each treatment group (A = control, B = vitexin, C = daunorubicin, and D = combination treatment). All treatments significantly increased apoptosis compared with the control (*p < 0.05, **p < 0.01, ***p < 0.001, ****p < 0.0001).

Following 48 hours of treatment, both NB-4 and MOLT-4 leukemic cells exhibited significantly increased apoptosis in response to vitexin, daunorubicin, and their combination. In NB-4 cells (Fig. 3A), vitexin and daunorubicin individually increased apoptosis to 42.82% and 45.53%, respectively, and the combination further increased apoptosis to 55.00%, a modest but statistically significant enhancement. In MOLT-4 cells (Fig. 3B), vitexin and daunorubicin elevated apoptosis to 40.0% and 42.03%, respectively, while the combination treatment produced a comparable level of 42.05%, indicating an additive rather than synergistic effect. These results demonstrate that vitexin enhances daunorubicin-induced apoptosis in NB-4 cells, whereas the interaction in MOLT-4 cells demonstrated an additive effect.

### Vitexin and candidate protein interaction using in silico bioinformatic tools

**Fig. 4:**
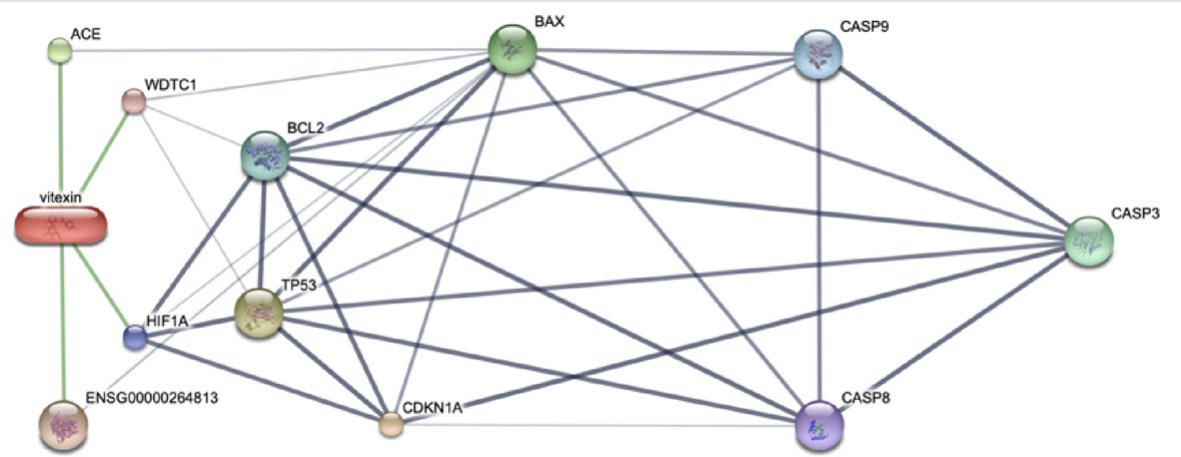
In silico bioinformatic analysis of vitexin’s interaction with apoptosis-related proteins. This figure presents a confidence view generated from the STITCH database, illustrating the predicted interactions between vitexin and candidate target proteins involved in programmed cell death (apoptosis). Chemical-protein interactions are depicted by green lines, while protein-protein interactions are represented by grey lines. The thickness of each line signifies the strength of the association, with thicker lines indicating stronger confidence in the interaction.

A computational investigation of the potential mechanisms by which vitexin induces apoptosis, an in silico bioinformatics analysis revealed a strong interaction between vitexin and hypoxia-inducible factor 1-alpha (HIF-1α) (Fig. 4). HIF-1α plays a pivotal role in cellular responses to hypoxia and is frequently overexpressed in cancer, contributing to tumor progression, metastasis, and therapy resistance. Notably, HIF-1α has the capacity to upregulate the expression of the anti-apoptotic protein Bcl-2, thereby enhancing cell survival. This interaction suggests a protective mechanism by which HIF-1α may promote cell survival by inhibiting programmed cell death. The predicted downstream effects of this interaction also hint at possible pathways leading to the activation of caspase-3, a key executioner in the caspase cascade. These identified candidate target proteins, specifically HIF-1α and Bcl-2, and caspase-3, will serve as focal points for subsequent experimental investigations aimed at elucidating the molecular mechanisms through which vitexin may induce apoptotic signaling pathways.

### The effect of vitexin on target gene expression

**Fig. 5:**
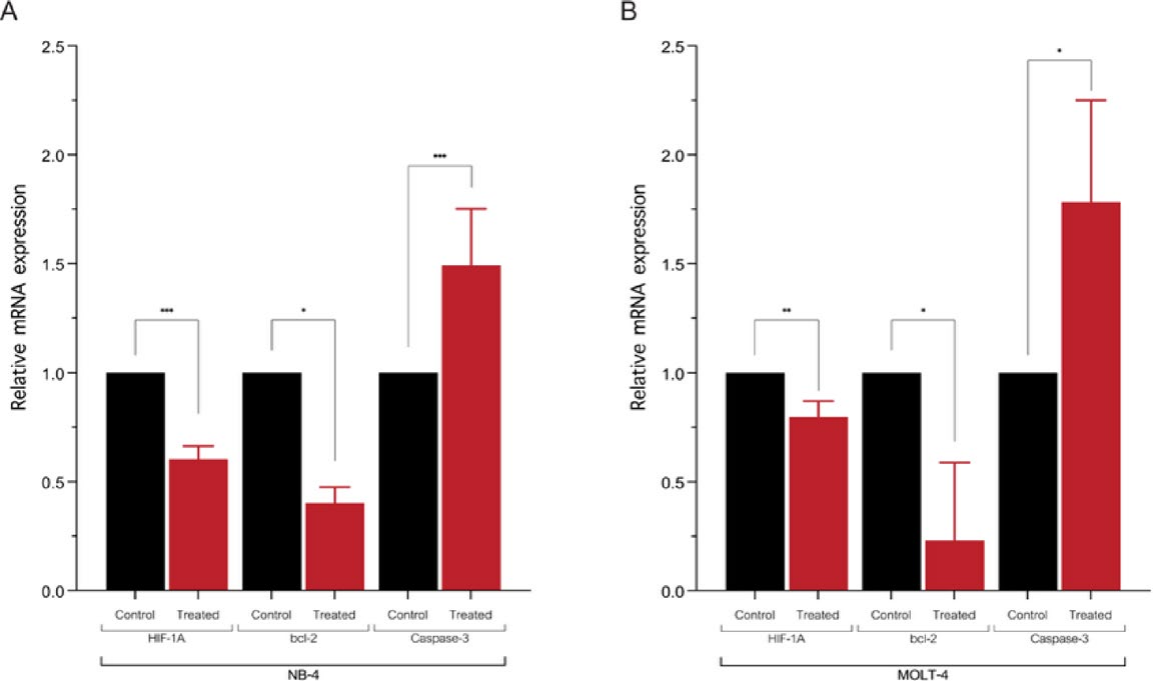
The effect of vitexin on apoptosis-related gene expression in Leukemic Cell Lines. This figure illustrates the expression levels of candidate target genes, including *HIF-1α*,* bcl-2*, and *caspase-3*, in NB-4 (**A**) and MOLT-4 (**B**) leukemic cell lines following vitexin treatment, as quantified by RT-qPCR. This technique allowed for the precise quantification of mRNA levels, facilitating a comparative assessment of gene expression. (*p < 0.05, **p < 0.01, ***p < 0.001, ****p < 0.0001).

The impact of vitexin on the expression of specific apoptosis-related genes was investigated using RT-qPCR. This method allowed for the quantification of *HIF-1α*,* Bcl-2*, and *caspase-3* gene expression. After 48 hours of vitexin treatment, RT-qPCR results revealed a significant reduction in the expression levels of both *HIF-1α* and the anti-apoptotic gene *bcl-2* in both NB-4 and MOLT-4 leukemic cell lines. Conversely, the expression level of *caspase-3*, a key executioner in the apoptotic cascade, was markedly elevated in both cell lines (Fig. 5). These findings suggest that vitexin possesses a dual capacity in the inhibition of anti-apoptotic, while simultaneously promoting the activation of pro-apoptotic genes. This combined action ultimately leads to the activation of apoptotic signaling pathways, thereby increasing the susceptibility of leukemic cells to undergo programmed cell death. These results have significant implications for potential therapeutic interventions in leukemia treatment.

### Determination of protein expression by western blot analysis

The effect of vitexin on protein expression levels of HIF-1α and caspase-3 was evaluated in both NB-4 and MOLT-4 leukemic cell lines using western blot analysis. This assessment was conducted after a 48 hours of treatment period with the IC_50_ concentration of vitexin.

**Fig. 6:**
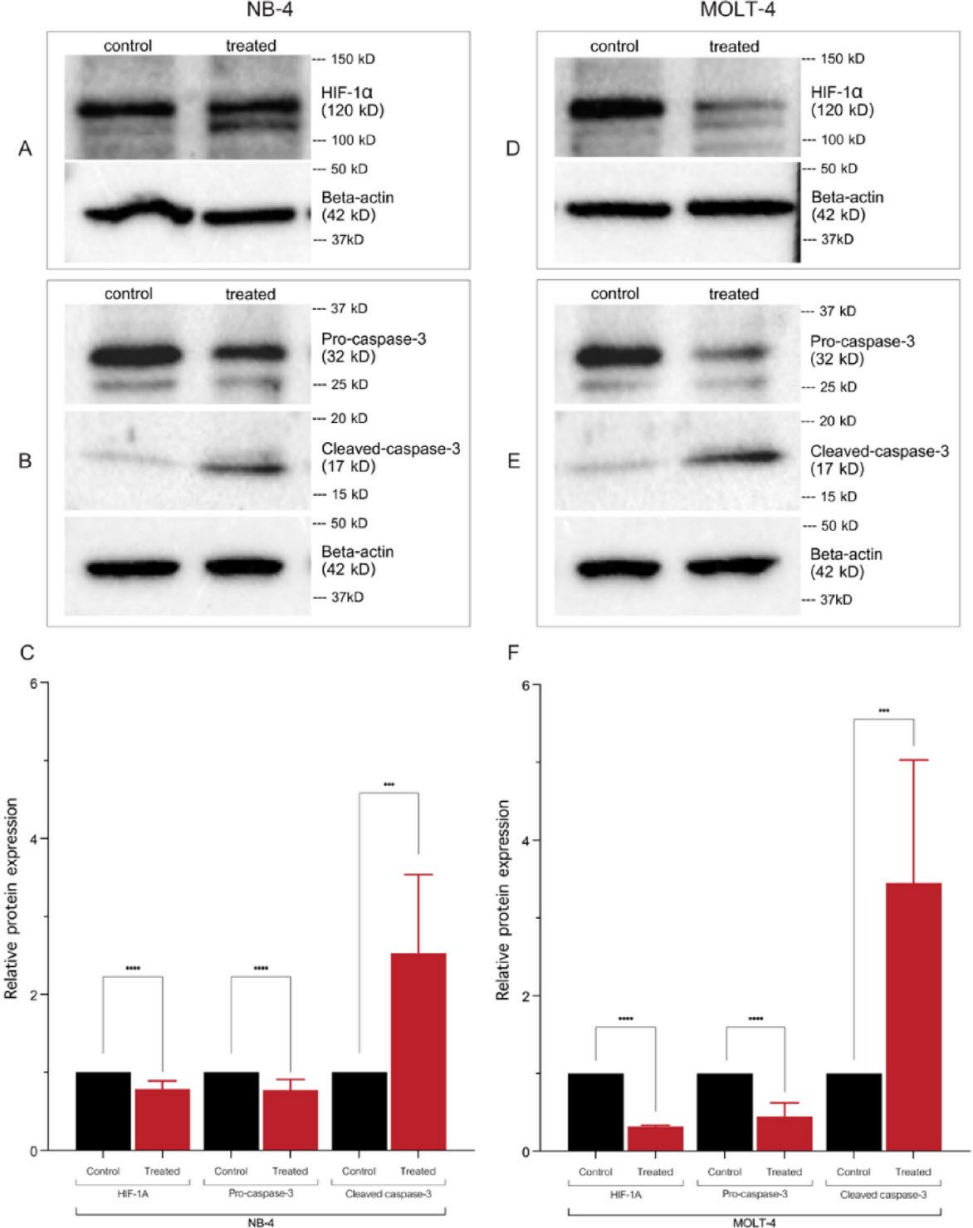
Western blot analysis of apoptosis-related proteins in NB-4 (**A**–**C**) and MOLT-4 (**D**–**F**) leukemic cell lines following 48 hours of treatment with the IC_50_ vitexin concentration. HIF-1α, pro-caspase-3, and cleaved-caspase-3 were analyzed, with β-actin as the loading control. The quantification of relative protein expression normalized to β-actin is shown in the bar graphs (**C** and **F**). Full-length uncropped blots corresponding to the results are provided in the supplementary figures. (*p < 0.05, **p < 0.01, ***p < 0.001, ****p < 0.0001).

The findings demonstrate that vitexin significantly decreased the expression of both HIF-1α and pro-caspase-3, conversely increased the level of cleaved caspase-3 protein (Fig. 6). The presence of cleaved caspase-3 specifically indicates the activation of caspase-dependent apoptotic pathways, which are crucial for programmed cell death. These results strongly suggest that vitexin effectively initiates apoptosis in both NB-4 and MOLT-4 leukemic cell lines, as evidenced by clear differences observed between the treated and corresponding control groups (Fig. 6C, F).

### The effect of vitexin and combination treatment on inducing apoptosis in bone marrow-derived leukemic cells

**Fig. 7:**
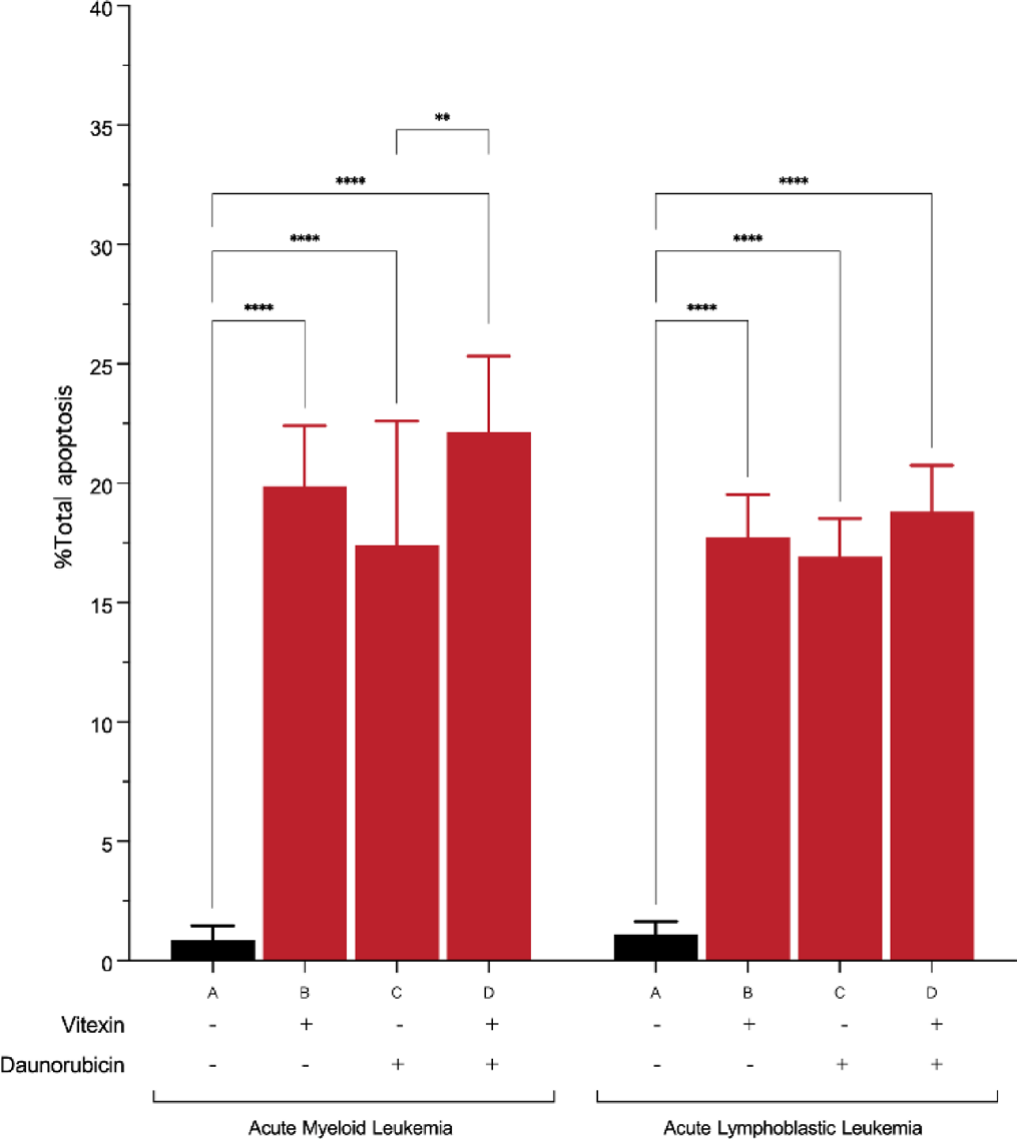
Bone marrow-derived leukemic cells were treated with an IC_50_ concentration of vitexin, daunorubicin, and a combination for 48 hours (A = control, B = vitexin, C = daunorubicin, and D = combination treatment). Apoptosis was assessed by flow cytometry using CD45, annexin V, and propidium iodide (PI) staining. Bar graph comparing the percentage of total apoptotic cells in AML and ALL across different treatment groups. (*p < 0.05, **p < 0.01, ***p < 0.001, ****p < 0.0001)

Bone marrow-derived leukemic cells obtained from patients diagnosed with acute leukemia were treated with an IC_50_ concentration of vitexin, daunorubicin, and their combination for a duration of 48 hours. Subsequently, the overall percentage of apoptosis was quantitatively evaluated using flow cytometry (Fig. 7).

The findings reveal that in AML, the control group exhibited a minimal level of apoptosis, approximately 0.90%. Treatment with vitexin, daunorubicin, and their combination resulted in significant increases in apoptosis rates, measuring 19.88%, 17.40%, and 22.15%, respectively. In the case of ALL, the control group displayed an apoptosis baseline of approximately 1.11%. Corresponding treatments with vitexin, daunorubicin, and their combination led to notable increases in apoptosis, with rates of 17.73%, 16.90%, and 18.82%, respectively.

These findings indicate that vitexin and daunorubicin each induced apoptosis effectively in bone marrow-derived leukemic cells from both AML and ALL patients. In AML samples, the combination treatment further increased the apoptotic level that was significantly higher than either single agent (*p* < 0.01). By contrast, in ALL samples, while the combination treatment resulted in the highest numerical percentage of apoptosis, the differences between vitexin, daunorubicin, and the combination did not reach statistical significance. These results suggest that vitexin enhances daunorubicin-induced apoptosis in AML, whereas in ALL, the combination effect is observed only as a non-significant trend.

## Discussion

The study demonstrated the investigation of the therapeutic potential of vitexin, a flavonoid found in various plants, including mung bean, known for its established antioxidant and anticancer properties^24,28,32^. Our findings compellingly demonstrate the efficacy of vitexin as an apoptosis-inducing agent in leukemic cells. The observed significant dose- and time-dependent cytotoxic effects in both NB-4 (representing acute myeloid leukemia) and MOLT-4 (representing acute lymphoblastic leukemia) cell lines provide substantial evidence that this natural compound holds promise as an alternative or adjunct to current acute leukemia treatment strategies. This aligns with existing literature suggesting that natural compounds can engage multiple cellular pathways, thereby potentially enhancing therapeutic efficacy while mitigating the adverse effects commonly associated with conventional chemotherapeutic drugs. Flow cytometry analysis confirmed the pro-apoptotic activity of vitexin in both leukemic models, increasing apoptosis to 42.82% in NB-4 cells and 40.0% in MOLT-4 cells following 48 hours of treatment. These results highlight the ability of vitexin to initiate programmed cell death and provide a mechanistic basis for evaluating its interaction with daunorubicin in combination treatment. In addition to the effects of vitexin alone, we further evaluated the apoptotic response to combination treatment in NB-4 and MOLT-4 cells. In NB-4 cells, the combination of vitexin and daunorubicin induced a significantly higher level of apoptosis compared with daunorubicin alone, suggesting a synergistic enhancement that is consistent with the CI analysis. In contrast, in MOLT-4 cells, the combination treatment resulted in apoptosis levels comparable to those observed with each single agent, reflecting an additive rather than synergistic interaction, in agreement with the CI value of 1.0.

Mechanistically, apoptosis is regulated by a multifaceted network of signaling pathways, modulated by an array of pro-apoptotic and anti-apoptotic proteins^10,12^. The findings revealed that vitexin downregulated anti-apoptotic Bcl-2 and HIF-1α while concurrently increasing pro-apoptotic caspase-3 expression, confirming that the regulation of the HIF-1α/Bcl-2/Caspase-3 ultimately contributed to programmed cell death in leukemic cells. Although this pathway is well documented, our results reinforce the ability of vitexin to modulate these apoptotic regulators in acute leukemic cells.

In primary bone marrow-derived samples, vitexin induced apoptosis in both AML and ALL patient cells. Notably, the combination demonstrated a significantly greater apoptotic response in AML samples, whereas the increase observed in ALL samples did not reach statistical significance. These results suggest that vitexin may preferentially enhance daunorubicin-mediated apoptosis in myeloid leukemias. It should also be emphasized that the combination experiments were designed as an early mechanistic assessment to determine whether vitexin could potentiate daunorubicin-induced apoptosis at the cellular level, rather than to model or modify existing clinical induction regimens.

Several limitations should be acknowledged. First, vitexin requires relatively high in vitro concentrations to achieve cytotoxic and pro-apoptotic effects, reflecting its poor solubility and restricted membrane permeability^33^, which is consistent with findings from previous studies on other cancer models^20^. Notably, PBMCs remained unaffected at IC_50_ equivalent concentrations, indicating that the effects observed were unlikely to result from nonspecific toxicity. However, the concentrations are not clinically achievable in vivo. Therefore, the present findings should be interpreted mechanistically rather than translationally. It is also important to clarify that the combination experiments were conducted as an early mechanistic evaluation, aiming to determine whether vitexin could enhance daunorubicin-induced apoptosis at the cellular level, rather than to model or modify existing clinical induction regimens. In addition, although NB-4 and MOLT-4 provided representation of myeloid and lymphoid leukemic cells, the use of two cell lines is not able to capture the biological heterogeneity of acute leukemia. The addition of AML (such as HL-60, THP-1) and ALL models (such as CCRF-CEM, Jurkat), as well as daunorubicin-resistant models, would further strengthen the investigation of the results. The four-point concentration range used in this study allowed us to establish an initial inhibitory profile for vitexin, a more detailed dose-response curve may further enhance accuracy. Increasing the number of dose points across a broader range would strengthen future IC_50_ determination and provide a more comprehensive pharmacodynamic assessment. CI analysis relied on a single combination dose offered preliminary data but does not represent full pharmacodynamic characterization. A more comprehensive dose-response assessment, together with a full Chou-Talalay synergy design, will be required to confirm the interaction profile between vitexin and daunorubicin.

Furthermore, although apoptosis was evaluated in 15 AML and 15 ALL primary bone marrow samples, the single dose used limited the ability to assess dose-response or quantify drug interactions, and the enhancement observed with the combination should be interpreted as preliminary rather than definitive evidence of synergy. Mechanistically, the present work focused primarily on the downstream apoptotic regulator within the HIF-1α/Bcl-2/Caspase-3. The upstream pathways modulating HIF-1α, such as PI3K/AKT, MAPK/ERK, or mTOR, were not evaluated, and it remains unclear whether HIF-1α downregulation occurs through which mechanisms. Finally, in vivo studies will be necessary to clarify the pharmacokinetics, toxicity profile, and therapeutic relevance of vitexin, and future investigations may provide benefit from exploring combinations with clinically relevant agents such as Bcl-2 inhibitors (e.g., venetoclax) to expand mechanistic insight.

In conclusion, this study demonstrates that vitexin exerts clear pro-apoptotic activity in both leukemic cell lines and primary bone marrow-derived leukemic cells. Vitexin was able to induce apoptosis across myeloid and lymphoid lineages, and in NB-4 cells, it further enhanced the apoptotic effect of daunorubicin, consistent with the synergistic interaction observed in viability assays. In MOLT-4 cells and ALL primary samples, the combination produced an additive rather than synergistic response, underscoring lineage-specific differences in drug interaction. Mechanistically, vitexin modulated key apoptotic regulators within the HIF-1α/Bcl-2/Caspase-3, providing evidence of its ability to influence pathways relevant to leukemic cell survival.

While the findings highlight a potential role for vitexin as a pro-apoptotic agent and as a possible enhancer of daunorubicin activity, the study is exploratory and mechanistic. The high concentrations required, limited cell line representation, and single dose used in both cell lines and patient samples indicate that further work is necessary. Future studies incorporating comprehensive dose-response analyses, full synergy models, upstream pathway investigation, and in vivo validation will be essential to clarify the therapeutic relevance of vitexin and to determine whether it may serve as a feasible adjuvant to conventional chemotherapy in acute leukemia.

## Materials and methods

### Ethical statement

The human research and experiments were approved by the Siriraj Institutional Review Board (SIRB, MU-MOU CoA No. 085/2025) and conducted according to relevant national and international guidelines to maintain patient data confidentiality and obtain informed consent from all participants. All methods were performed in accordance with the relevant guidelines and regulations, including the Declaration of Helsinki, the Belmont Report, CIOMS Guidelines, and the International Conference on Harmonization in Good Clinical Practice (ICH-GCP).

### Reagent and chemical

Vitexin compound was provided by Professor Dr.Attasak Jaree, Department of Chemical Engineering, Faculty of Engineering, Kasetsart University. A 200 mM stock solution of vitexin was prepared by dissolving it in Dimethyl Sulfoxide (DMSO) and stored at -20 °C. 3-(4,5-dimethylthiazol-2-yl)-2,5-diphenyl tetrazolium bromide (MTT) was obtained from Invitrogen (Waltham, MA, USA). The Annexin V-FITC Apoptosis Detection Kit with Propidium Iodide (PI), Anti-CD45 antibody was purchased from BD Biosciences (Palo Alto, CA, USA). Luna^®^ Universal qPCR Master Mix was acquired from New England Biolabs (Ipswich, MA, USA). Antibodies against total beta-actin, caspase-3, and HIF-1α were obtained from Cell Signaling Technology (Danvers, MA, USA). Ultra-pure water was utilized for the preparation of all reagents and in all experimental procedures.

### Cell culture

The NB-4 (acute myeloid leukemia) and MOLT-4 (acute lymphoblastic leukemia) cell lines were acquired from Cell Line Services (Eppelheim, Germany). Leukemic cells were cultured in RPMI-1640 medium supplemented with 10% fetal bovine serum (FBS) and 1% penicillin-streptomycin (Gibco Life Technologies, Waltham, MA, USA) as an antibiotic. Cells were cultured in a humidified atmosphere containing 5% CO_2_ at 37˚C and subcultured every 3 days.

### Sample collection and preparation

Peripheral blood mononuclear cells (PBMCs) were collected from 40 healthy donors (MU-MOU CoA No. 085/2025) and isolated using LymphoprepTM (Alere Technologies AS, Oslo, Norway) according to the manufacturer’s instructions. Briefly, EDTA blood was diluted 1:1 with phosphate-buffered saline (PBS). LymphoprepTM was then added to 50 ml conical tubes, and the diluted blood was carefully layered on top of the LymphoprepTM solution. Cell separation was achieved by centrifugation at 800 g for 20 min without decline acceleration. The resulting PBMC layer was then carefully collected and washed twice with RPMI-1640 medium before being used for subsequent experiments.

A total of 30 bone marrow samples were collected from acute leukemia patients, with equal distribution between those diagnosed with acute myeloid leukemia and acute lymphoblastic leukemia (MU-MOU CoA No. 085/2025). Samples were obtained from the Division of Hematology, Department of Medicine, Siriraj Hospital, Mahidol University, using whole EDTA bone marrow for subsequent experiments.

### Measurement of cell cytotoxicity by using MTT

Leukemic cell lines NB-4 and MOLT-4, alongside normal peripheral blood mononuclear cells (PBMCs), were cultured in 96-well plates. NB-4 and MOLT-4 cells were seeded at a density of 1.5 × 10^4^ cells per well, while PBMCs were seeded at 1 × 10^5^ cells/well. Leukemic cells were then treated with varying concentrations of vitexin, as detailed in Table 1. Following incubation for 24 and 48 hours in a humidified 5% CO_2_ atmosphere at 37 °C, cell viability was assessed using the MTT assay. Briefly, 10 µL of a 5 mg/mL MTT solution was added to each well and incubated for 4 hours. The resulting formazan crystals were solubilized by adding 100 µL of 10% SDS in 0.01 M HCl, followed by overnight incubation. Absorbance was measured at 570 nm using a microplate reader with Gen5^™^ analysis software (BioTek Instruments, Inc., Winooski, VT, USA). Then, the half-maximal inhibitory concentration (IC_50_) was calculated for subsequent experiments.

**Table 1:** The treatment protocol - various concentrations of compounds or drugs used in the experiment.

Compounds/drugs	Concentration (µM)				Incubation period
Vitexin	0	500	1000	1500	24 and 48 hours
Daunorubicin	0	0.2	0.4	0.8	24 and 48 hours

$$\% {\mathrm{Cell}}\;{\mathrm{viability}}=\frac{{{\mathrm{O}}{{\mathrm{D}}_{570{\mathrm{nm}}}}\;{\mathrm{of}}\;{\mathrm{sample}}}}{{{\mathrm{O}}{{\mathrm{D}}_{570{\mathrm{nm}}}}\;{\mathrm{of}}\;{\mathrm{control}}}} \times 100$$

### Combination drug investigation

To evaluate potential synergistic effects of vitexin, leukemic cell lines were treated with their predetermined IC_50_ concentration of vitexin, both alone and in combination with daunorubicin. Cell viability was assessed using the MTT assay as described above. The combination index (CI) was calculated using CompuSyn software to quantitatively analyze the nature of the drug interactions (synergistic, additive, or antagonistic). The interaction between vitexin and daunorubicin was evaluated using the Chou-Talalay method. CI values were interpreted as follows, CI < 1 indicates synergism, CI = 1 indicates an additive effect, and CI > 1 indicates antagonism^34^.

### Detection of apoptosis by flow cytometry with Annexin V/PI staining

Apoptosis was detected by assessing the externalization of phosphatidylserine (PS), a phospholipid typically located on the inner leaflet of healthy cell membranes. This process involves the use of Annexin V-fluorescein isothiocyanate (FITC), which specifically binds to externalized PS, indicating early-stage apoptosis. In conjunction with propidium iodide (PI), a nucleic acid stain, allows for the identification of cells with compromised membrane integrity, characteristic of late-stage apoptosis, as PI can bind to double-stranded DNA within the cells.

NB-4 and MOLT-4 leukemic cells were cultured in 24-well plates at a density of 1 × 10^5^ cells per well. Cells were treated with the IC_50_ concentrations of vitexin, daunorubicin, or the combination of both agents for 48 hours in a humidified 5% CO_2_ atmosphere at 37 °C. After treatment, cells were collected by centrifugation at 1,500 rpm for 5 minutes at 4 °C and washed twice with phosphate-buffered saline (PBS). The cell pellets were resuspended in 100 µL of 1X binding buffer and transferred to flow cytometry tubes. Annexin V-FITC (2 µL) and PI (2 µL) were added to each tube, followed by a 15 minutes of incubation at room temperature in the dark. Subsequently, 400 µL of 1X binding buffer was added. Apoptotic cells were analyzed using a FACSCanto II flow cytometer, and data were quantified using FACSDiva software (BD Biosciences, San Jose, CA, USA).

### Prediction of protein-chemical interactions using bioinformatic tools

Bioinformatic analysis was performed using the STITCH database (http://stitch.embl.de/) to investigate the potential interactions and correlations between natural compounds and proteins, thereby identifying novel candidate proteins or potential therapeutic targets. Vitexin was used as a keyword to search for apoptotic-related proteins, with a medium confidence level (0.400) selected for interaction analysis.

### Gene expression by reverse transcription quantitative PCR (RT-qPCR)

The expression levels of apoptosis-related genes, including *hypoxia-inducible factor-1 alpha (HIF-1α)*, *Bcl-2*, and *caspase-3*, were quantified using RT-qPCR. Leukemic cells were treated with an IC_50_ concentration of vitexin for 48 hours in a humidified 5% CO_2_ atmosphere at 37 °C. Total RNA was extracted using Genezol^™^ reagent according to the manufacturer’s protocol. RNA concentration was determined using a Nanodrop 2000, and RNA was subsequently reverse-transcribed into complementary DNA (cDNA) using a reverse transcriptase enzyme. DNA levels were quantified using SYBR Green, a double-stranded DNA-binding dye. The RT-qPCR master mix contained DNA polymerase, specific primers (Table 2), SYBR Green dye, dNTPs, and buffer. Real-time PCR was performed on a real-time PCR detection system, with glyceraldehyde 3-phosphate dehydrogenase (GAPDH) serving as an internal control for normalization. Gene expression levels were quantified relative to the internal control.

**Table 2:** Primer sequence of target genes.

Name	Sequence (5’ ◊ 3’)
*HIF-1α*	Forward primer: GTCTGAGGGGACAGGAGGAT Reverse primer: CTCCTCAGGTGGCTTGTCAG
*Bcl-2*	Forward primer: ATGTGTGTGGAGAGCGTCAA Reverse primer: GCCGTACAGTTCCACAAAGG
*Caspase-3*	Forward primer: TTCAGAGGGGATCGTTGTAGAAGTC Reverse primer: CAAGCTTGTCGGCATACTGTTTCAG

### Determination of protein expression by western blot analysis

The expression levels of apoptosis-related proteins, specifically hypoxia-inducible factor-1 alpha (HIF-1α) and caspase-3, were examined by western blot analysis. Leukemic cells were treated with their IC_50_ concentration of vitexin for 48 hours and subsequently harvested for protein extraction. Protein extraction was performed using RIPA lysis buffer, followed by a 30 minutes of incubation on ice and centrifugation at 14,000 g for 10 min at 4 °C to obtain the protein supernatant. Extracted proteins were separated by sodium dodecyl sulfate-polyacrylamide gel electrophoresis (SDS-PAGE) and then blotted onto polyvinylidene fluoride (PVDF) membranes for 2 hours. Membranes were blocked with either 5% non-fat milk or 5% (w/v) bovine serum albumin (BSA) in Tris-buffered saline containing 0.1% Tween 20 (pH 7.4) at room temperature for 2 hours. Following blocking, membranes were incubated overnight with primary antibodies specific for caspase-3, HIF-1α, and β-actin (as a loading control). Subsequently, blots were incubated with horseradish peroxidase (HRP)-conjugated secondary antibodies. Protein signals were visualized using enhanced chemiluminescence.

### Detection of apoptosis by flow cytometry with CD45 and Annexin V/PI staining

Leukemic cells within primary bone marrow samples were investigated using flow cytometry by measuring apoptosis percentages with methods similar to those for cell lines, with added components for accuracy and specificity. The process involved dual staining with Annexin V and PI, along with CD45 staining, which helps distinguish leukocytes and gate leukemic cells based on scatter profiles, allowing analysis of apoptosis within these populations. Bone marrow cells from AML and ALL patients were cultured at 5.0 × 10^5^ cells per well, treated with vitexin, daunorubicin, and a combination for 48 hours in a humidified 5% CO_2_ atmosphere at 37 °C. Post-treatment, cells were collected, washed, and resuspended in binding buffer, then stained with 2.5 µL of CD45-PerCP, 2 µL of Annexin V-FITC, and 2 µL of PI, incubated in the dark for 15 min. After adding binding buffer, cells were analyzed by FACSCanto II flow cytometry, with leukemic populations gated based on CD45 positivity, and apoptotic cells assessed using FACSDiva software.

### Statistical analysis

All experiments involving leukemic cell lines were performed in triplicate, while bone marrow samples were analyzed based on a predetermined sample size calculation. Mean values and standard deviations were calculated for all experimental results. Statistical analyses were conducted using a student’s t-test for comparisons between two groups and a one-way analysis of variance (ANOVA) for comparisons involving more than two groups. The analyses were carried out using GraphPad Prism version 10.2.3 (GraphPad Inc., San Diego, CA, USA). A p-value of less than 0.05 was considered statistically significant.

## Supplementary Information

Below is the link to the electronic supplementary material.

Supplementary Material 1

## Data Availability

All data generated or analyzed during this study are included in this published article and its supplementary information files.
