# Supplementary material for: Vitexin induces apoptosis and enhances daunorubicin efficacy in acute leukemia via modulation of the HIF-1α/Bcl-2/caspase-3 pathway
Source: Sci Rep. 2025 Dec 14;16:2992. doi: 10.1038/s41598-025-32789-y (PMC12827246; doi:10.1038/s41598-025-32789-y)
Supplement: Supplementary file 1 — Supplementary Material 1 [file 41598_2025_32789_MOESM1_ESM.pdf]

**Supplementary Information for**

# Vitexin induces apoptosis and enhances daunorubicin efficacy in acute leukemia via modulation of the HIF-1α/Bcl-2/Caspase-3 pathway

Chutiphong Jirawatpraphakorn, Dalina Tanyong, Attasak Jaree, and Weerapat Owattanapanich*

## Supplementary Information

**Uncropped Western blot images corresponding to Figure 6.**

The uncropped blots for HIF-1α, pro-caspase-3, cleaved caspase-3, and β-actin are presented below. These full-length blots were used to generate the cropped images shown in the main manuscript (Figure 6).

Each target protein was normalized to β-actin from the same blot (within-gel normalization), and relative protein expression was calculated by setting the control group to 1.0 and expressing the treated group relative to its matched control.

**The NB-4 cell line**

**HIF-1α in the NB-4 cell line**

Uncropped blot

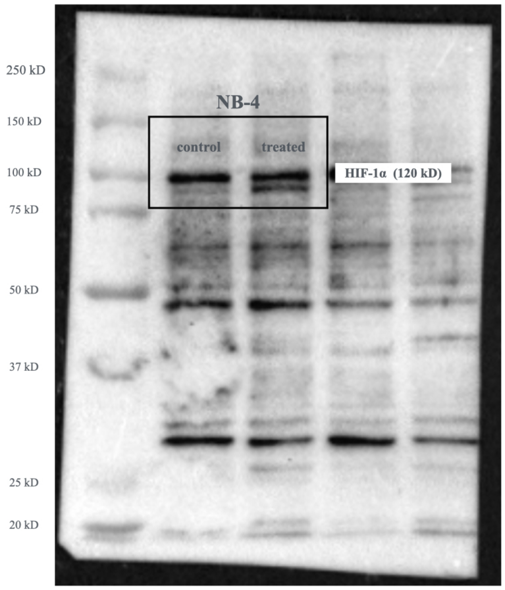


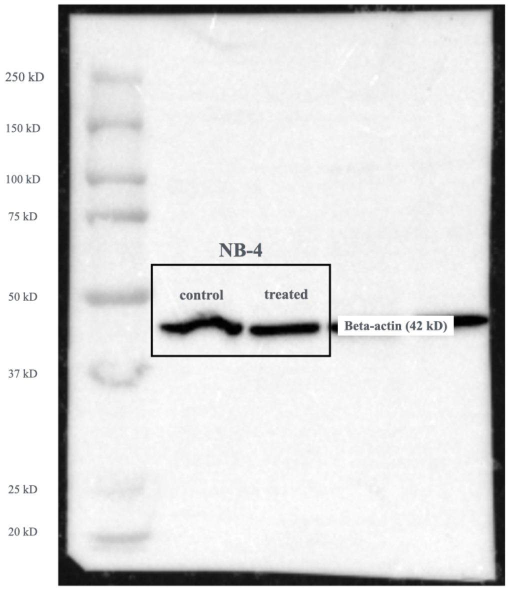


**Supplementary Figure S1A:** Full-length uncropped Western blot for HIF-1α in NB-4 cells**.**

Band intensity (HIF-1α)

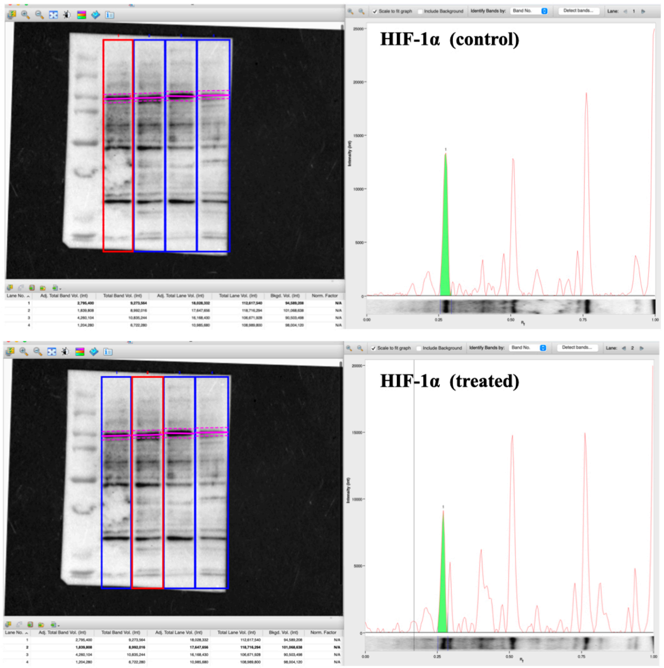


**Vitexin treatment in NB-4**

| Lane No. | Adj. Total Band Vol. (Int) | Total Band Vol. (Int) |
|---|---|---|
| 1 | 2,795,400 | 9,273,564 |
| 2 | 1,839,808 | 8,992,016 |
| 3 | 4,260,104 | 10,835,244 |
| 4 | 1,204,280 | 6,722,280 |

**Supplementary Figure S1B:** Band intensity for HIF-1α in NB-4 cells (normalized within gel).

Band intensity (Beta-actin)

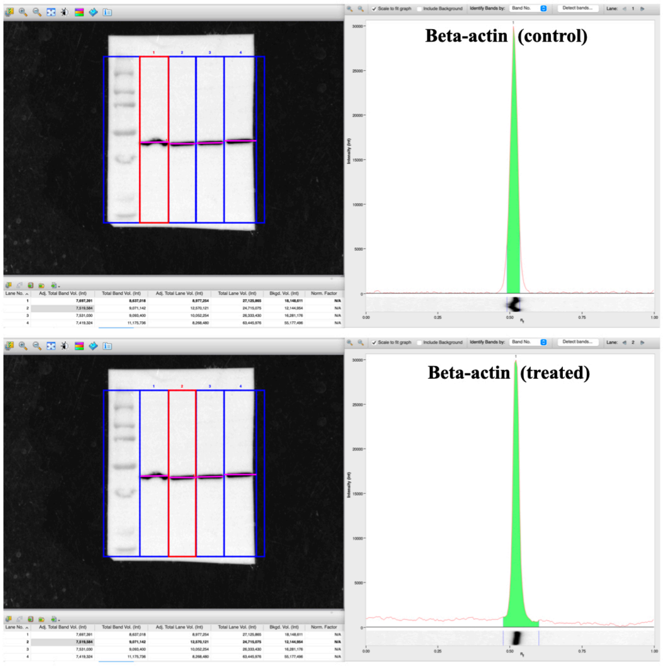


**Vitexin treatment in NB-4**

| Lane No. | Adj. Total Band Vol. (Int) | Total Band Vol. (Int) |
|---|---|---|
| 1 | 7,697,391 | 8,637,018 |
| 2 | 7,519,584 | 9,071,142 |
| 3 | 7,531,030 | 9,093,400 |
| 4 | 7,419,324 | 11,175,736 |

**Supplementary Figure S1C:** Band intensity for β-actin corresponding to the HIF-1α experiment in NB-4 cells

## Pro-caspase-3 in the NB-4 cell line

Uncropped blot

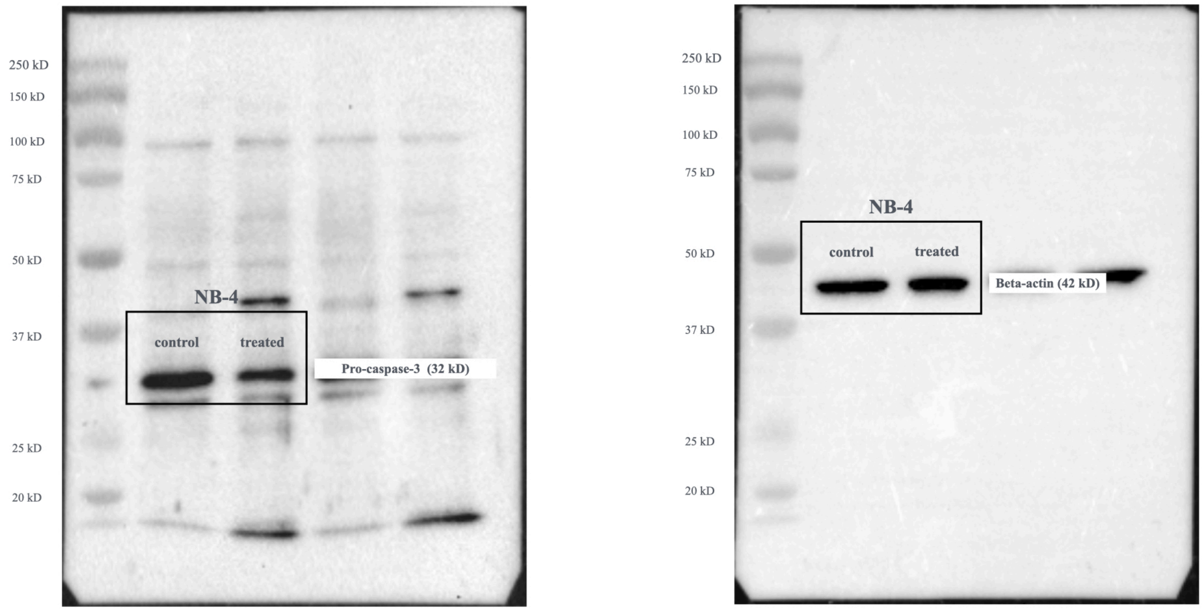


**Supplementary Figure S1D:** Full-length uncropped Western blot for pro-caspase-3 in NB-4 cells.

Band intensity (Pro-caspase-3)

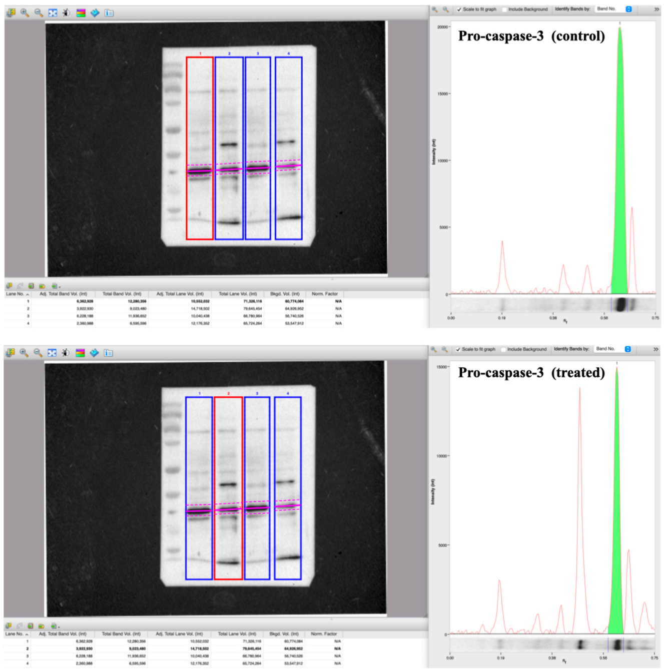


**Vitexin treatment in NB-4**

| Lane No. | Adj. Total Band Vol. (Int) | Total Band Vol. (Int) |
|---|---|---|
| 1 | 6,362,928 | 12,280,356 |
| 2 | 3,922,930 | 9,023,480 |
| 3 | 6,228,188 | 11,936,652 |
| 4 | 2,360,988 | 6,595,596 |

**Supplementary Figure S1E:** Band intensity for pro-caspase-3 in NB-4 cells (normalized within gel).

Band intensity (Beta-actin)

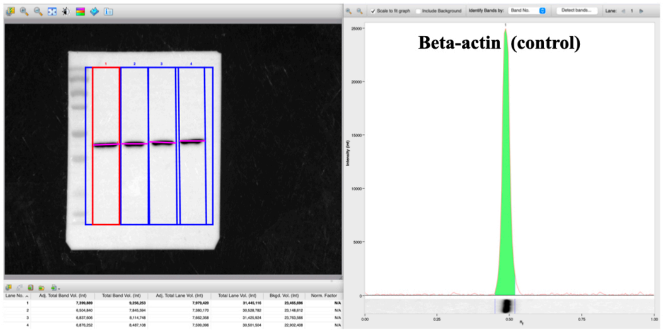


**Vitexin treatment in NB-4**

| Lane No. | Adj. Total Band Vol. (Int) | Total Band Vol. (Int) |
|---|---|---|
| **1** | **7,399,889** | **9,256,253** |
| 2 | 6,504,840 | 7,845,594 |
| 3 | 6,837,606 | 8,114,748 |
| 4 | 6,876,252 | 8,487,108 |

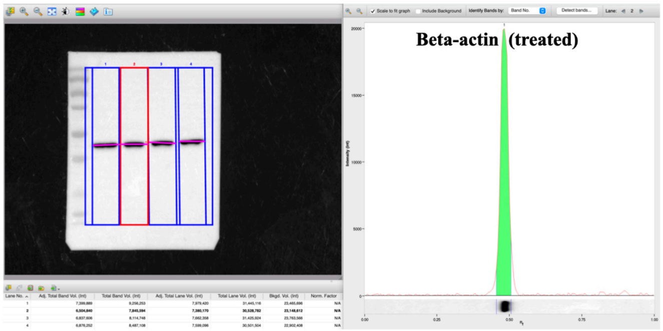


**Supplementary Figure S1F:** Band intensity for β-actin corresponding to the pro-caspase-3 experiment in NB-4 cells.

## Cleaved-caspase-3 in the NB-4 cell line

Uncropped blot

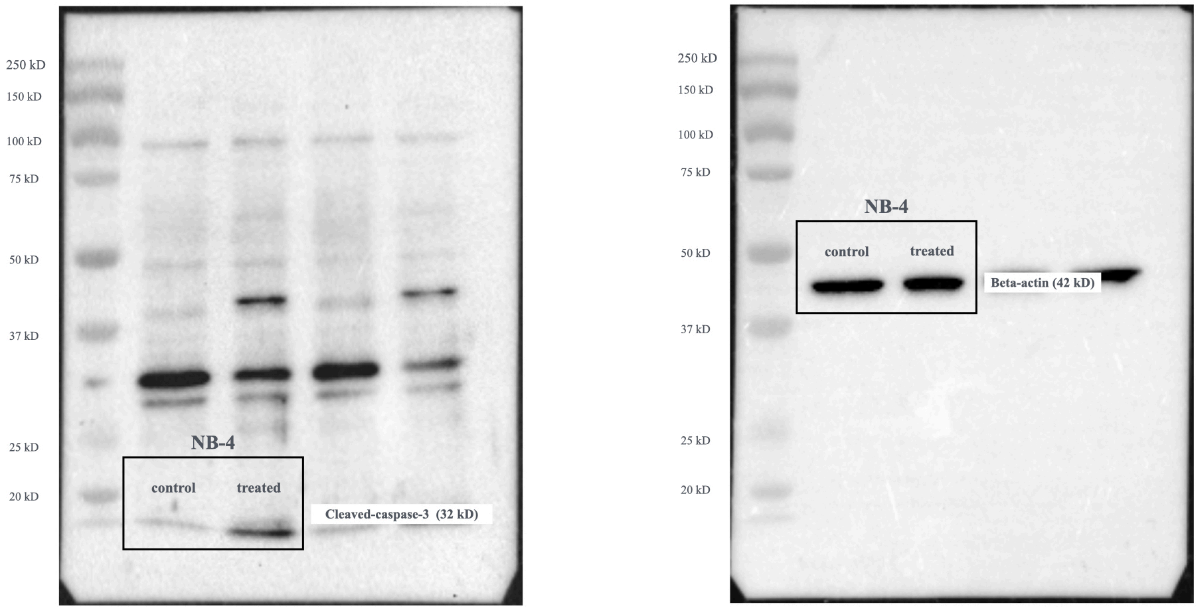


**Supplementary Figure S1G:** Full-length uncropped Western blot for cleaved caspase-3 in NB-4 cells.

Band intensity (Cleaved-Caspase-3)

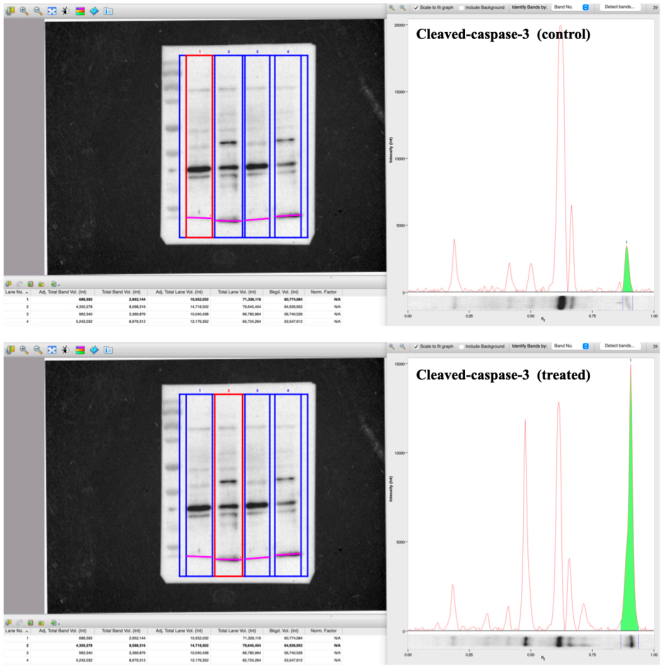


**Vitexin treatment in NB-4**

| Lane No. | Adj. Total Band Vol. (Int) | Total Band Vol. (Int) |
|---|---|---|
| 1 | 686,592 | 2,952,144 |
| 2 | 4,350,278 | 8,568,316 |
| **3** | **962,540** | **3,369,876** |
| 4 | 5,242,032 | 8,679,312 |

**Supplementary Figure S1H:** Band intensity for cleaved caspase-3 in NB-4 cells (normalized within gel).

Band intensity (Beta-actin)

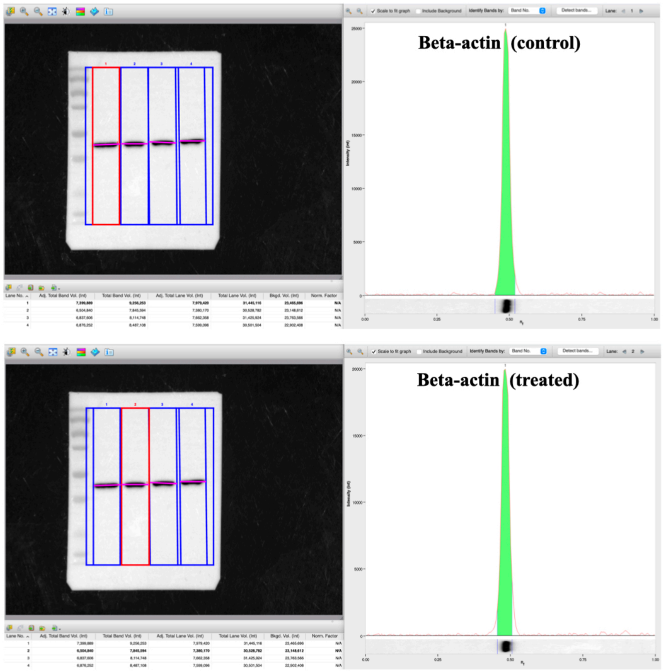


**Vitexin treatment in NB-4**

| Lane No. | Adj. Total Band Vol. (Int) | Total Band Vol. (Int) |
|---|---|---|
| **1** | **7,399,889** | **9,256,253** |
| 2 | 6,504,840 | 7,845,594 |
| 3 | 6,837,606 | 8,114,748 |
| 4 | 6,876,252 | 8,487,108 |

**Supplementary Figure S1I:** Band intensity for β-actin corresponding to the cleaved caspase-3 experiment in NB-4 cells.

**The MOLT-4 cell line**

**HIF-1α in the MOLT-4 cell line**

Uncropped blot

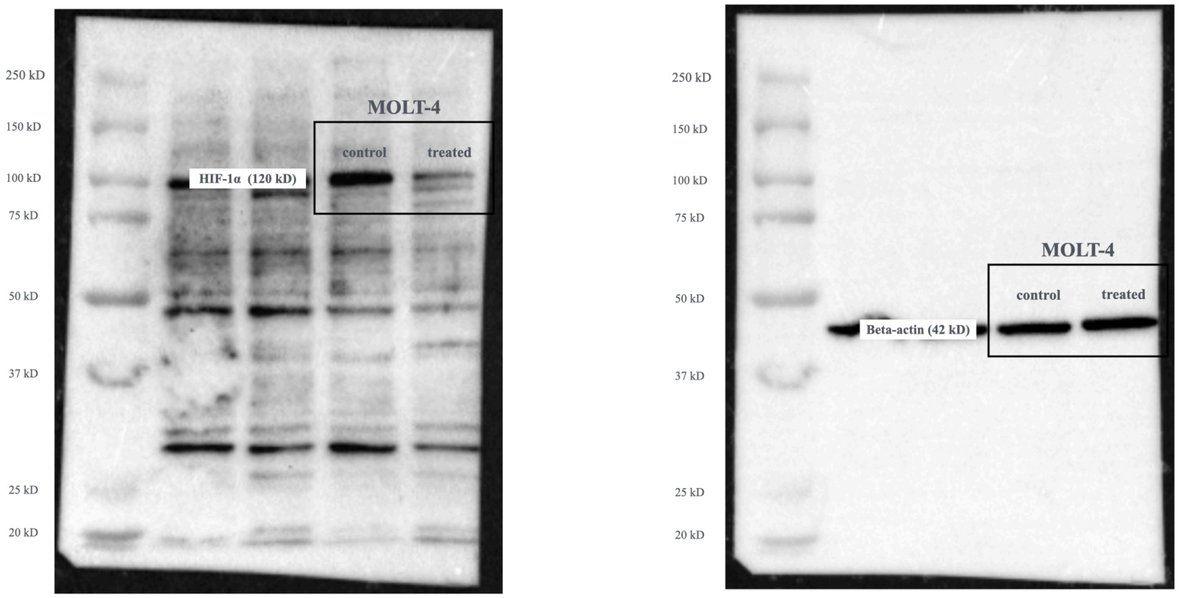


**Supplementary Figure S1J:** Full-length uncropped Western blot for HIF-1α in MOLT-4 cells**.**

Band intensity (HIF-1α)

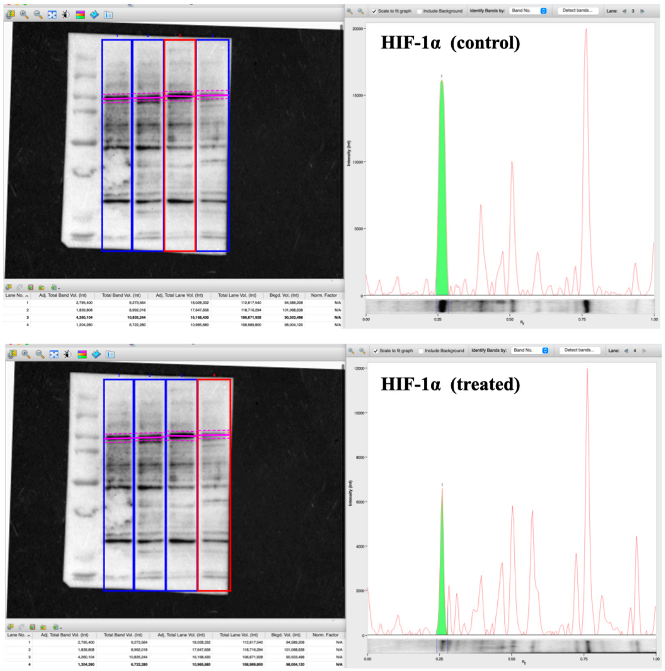


**Vitexin treatment in MOLT-4**

| Lane No. | Adj. Total Band Vol. (Int) | Total Band Vol. (Int) |
|---|---|---|
| 1 | **2,795,400** | **9,273,564** |
| 2 | 1,839,808 | 8,992,016 |
| 3 | 4,260,104 | 10,835,244 |
| 4 | 1,204,280 | 6,722,280 |

**Supplementary Figure S1K:** Band intensity for HIF-1α in MOLT-4 cells (normalized within gel).

Band intensity (Beta-actin)

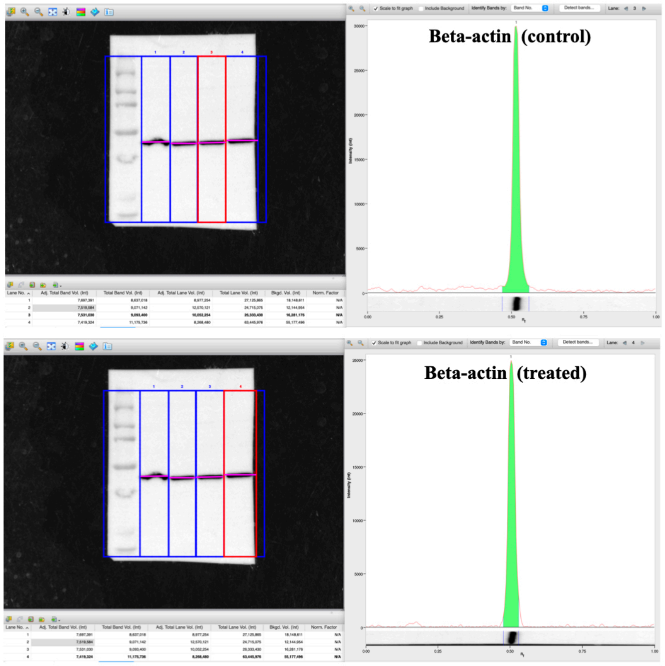


**Vitexin treatment in MOLT-4**

| Lane No. | Adj. Total Band Vol. (Int) | Total Band Vol. (Int) |
|---|---|---|
| **1** | **7,697,391** | **8,637,018** |
| 2 | 7,519,584 | 9,071,142 |
| 3 | 7,531,030 | 9,093,400 |
| 4 | 7,419,324 | 11,175,736 |

**Supplementary Figure S1L:** Band intensity for β-actin corresponding to the HIF-1α experiment in MOLT-4 cells

**Pro-caspase-3 in the MOLT-4 cell line**

Uncropped blot

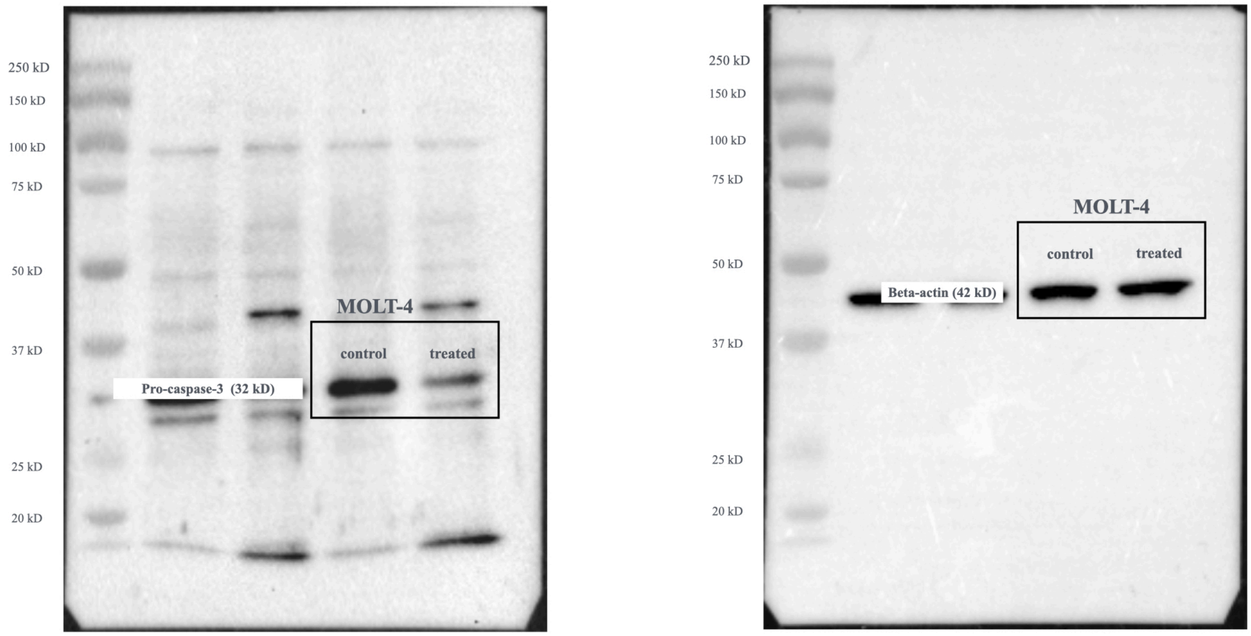


**Supplementary Figure S1M:** Full-length uncropped Western blot for pro-caspase-3 in MOLT-4 cells.

Band intensity (Pro-caspase-3)

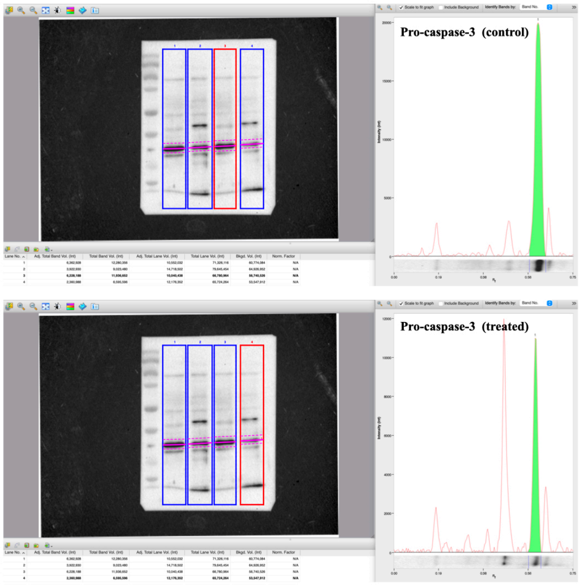


**Vitexin treatment in MOLT-4**

| Lane No. | Adj. Total Band Vol. (Int) | Total Band Vol. (Int) |
|---|---|---|
| 1 | 6,362,928 | 12,280,356 |
| 2 | 3,922,930 | 9,023,480 |
| **3** | **6,228,188** | **11,936,652** |
| 4 | 2,360,988 | 6,595,596 |

**Supplementary Figure S1N:** Band intensity for pro-caspase-3 in MOLT-4 cells (normalized within gel).

Band intensity (Beta-actin)

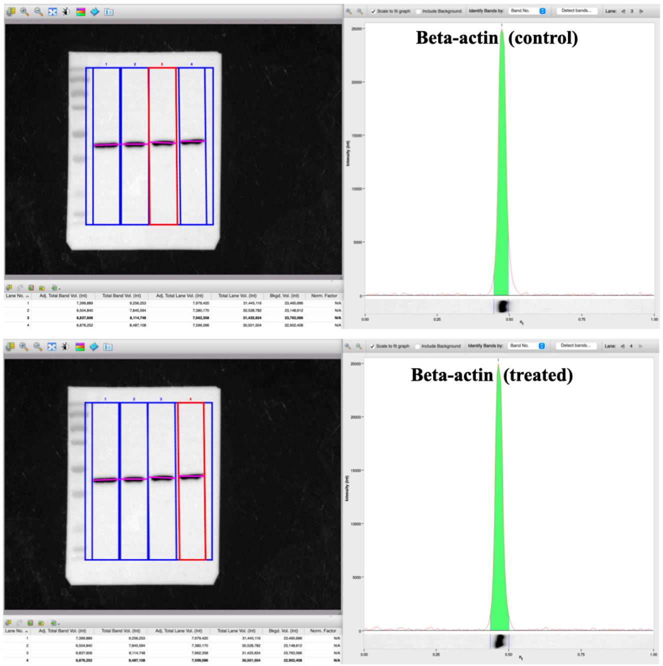


**Vitexin treatment in MOLT-4**

| Lane No. | Adj. Total Band Vol. (Int) | Total Band Vol. (Int) |
|---|---|---|
| **1** | **7,399,889** | **9,256,253** |
| 2 | 6,504,840 | 7,845,594 |
| 3 | 6,837,606 | 8,114,748 |
| 4 | 6,876,252 | 8,487,108 |

**Supplementary Figure S1O:** Band intensity for β-actin corresponding to the pro-caspase-3 experiment in MOLT-4 cells.

## Cleaved-caspase-3 in the MOLT-4 cell line

Uncropped blot

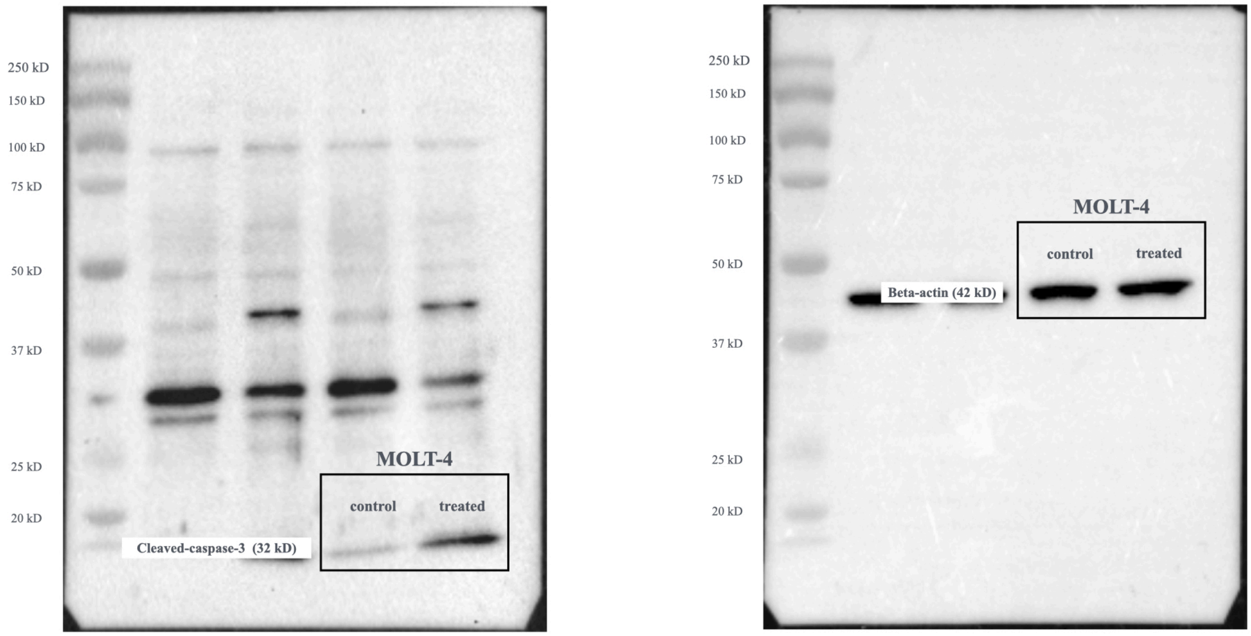


**Supplementary Figure S1P:** Full-length uncropped Western blot for cleaved caspase-3 in MOLT-4 cells.

Band intensity (Cleaved-Caspase-3)

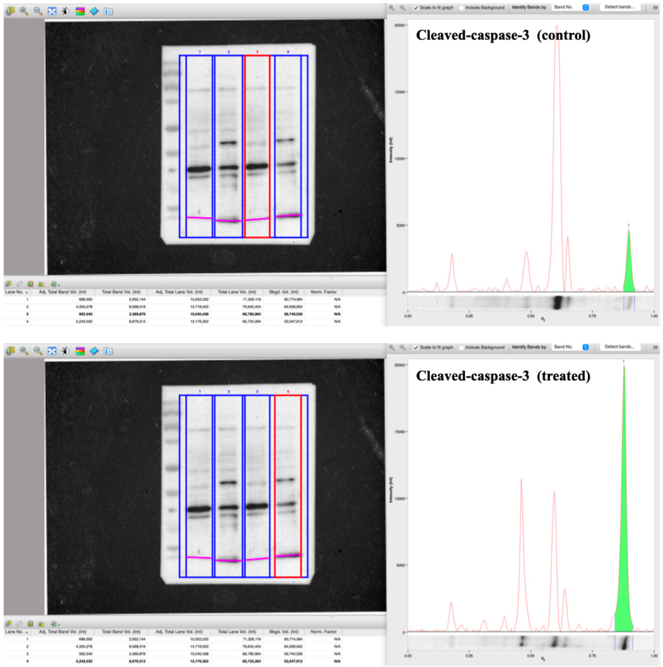


**Vitexin treatment in MOLT-4**

| Lane No. | Adj. Total Band Vol. (Int) | Total Band Vol. (Int) |
|---|---|---|
| 1 | 686,592 | 2,952,144 |
| 2 | 4,350,278 | 8,568,316 |
| **3** | **962,540** | **3,369,876** |
| 4 | 5,242,032 | 8,679,312 |

**Supplementary Figure S1Q:** Band intensity for cleaved caspase-3 in MOLT-4 cells (normalized within gel).

Band intensity (Beta-actin)

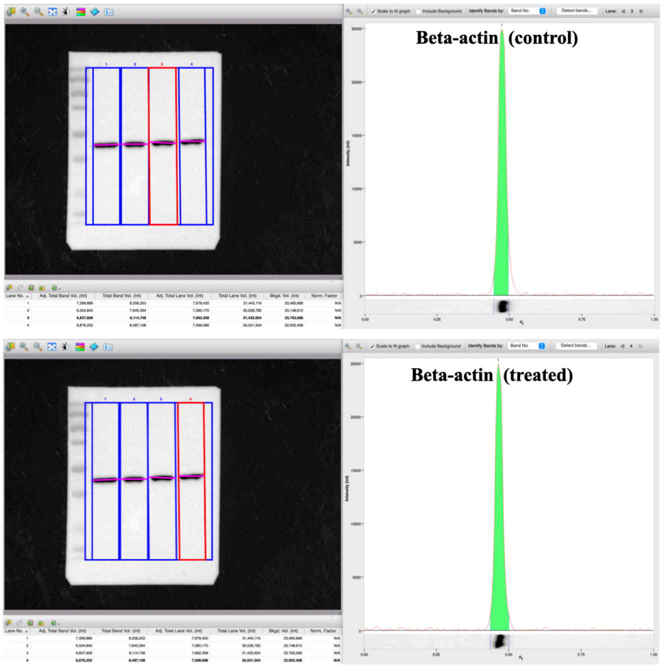


**Vitexin treatment in MOLT-4**

| Lane No. | Adj. Total Band Vol. (Int) | Total Band Vol. (Int) |
|---|---|---|
| **1** | **7,399,889** | **9,256,253** |
| 2 | 6,504,840 | 7,845,594 |
| 3 | 6,837,606 | 8,114,748 |
| 4 | 6,876,252 | 8,487,108 |

**Supplementary Figure S1R:** Band intensity for β-actin corresponding to the cleaved caspase-3 experiment in MOLT-4 cells.
